# Characterization of aloe vera gel incorporated unsaturated polyester resin jute-cotton fabric composites for enhanced biodegradability, flexibility, and insulation properties

**DOI:** 10.1016/j.heliyon.2024.e35261

**Published:** 2024-07-26

**Authors:** Md Anisur Rahman Dayan, Md Mahmudul Habib, Md Moslem Uddin, Mahmuda Khatun, Md Sahadat Hossain, Muhammad Abdur Rashid

**Affiliations:** a Textile Physics Division, Bangladesh Jute Research Institute, Dhaka, Bangladesh; b Technology Wing, Bangladesh Jute Research Institute, Dhaka, Bangladesh; c Product Development Division, Bangladesh Jute Research Institute, Dhaka, Bangladesh; d Institute of Glass and Ceramics Research and Testing, BCSIR, Dhaka, Bangladesh; e Department of Textile Engineering, DUET, Gazipur, Bangladesh

**Keywords:** Natural fibers, aloe vera gel, Characterization, Biodegradability, Flexibility, Insulation properties

## Abstract

In this research, Aloe Vera Gel (AVG) was incorporated into Unsaturated Polyester Resin (UPR) with jute-cotton union fabric to fabricate partially biodegradable composites. These composites were fabricated using a hand lay-up technique and characterized using Fourier Transform Infrared Spectroscopy (FTIR), Thermogravimetry Analysis (TGA), thermal conductivity measurements, water absorption tests, degradation assessments, cracking tests, and Universal Testing Machine (UTM) analysis. The study found that increasing the percentage of AVG in the composites led to a decrease in thermal conductivity, indicating improved insulation properties. Samples reinforced with AVG showed enhanced resistance to damage from iron nails, with reduced scratching and fiber displacement observed. However, the addition of AVG resulted in decreased thermal, mechanical, and water resistance properties compared to composites without AVG. FTIR analysis demonstrated interactions between AVG and the matrix materials. In degradation tests, composites subjected to an alkali environment (P^H^ = 11.96) showed the highest weight reduction (2.22 %) compared to those without AVG. Similarly, composites buried in soil exhibited greater weight loss (2.38 %) than their counterparts lacking AVG. Overall, the developed composite's reduced heat transfer rate suggests its potential application as an insulating material in environments such as rural poultry housing and the automotive industry.

## 1 Introduction

The growing interest in environmental concerns and sustainability, many researchers were focused on natural fibre-based polymer composites for several industrial and domestic applications for increasing the usage of natural fibres [1,2]. Though the synthetic fibres reinforced polymer composites have good mechanical properties in comparison to the natural fibres but these fibres are very expensive, non-biodegradable and not good for the environment [3,4]. The main sources of natural fibres are collected from plants or animals. Jute and cotton are the natural fibres which comes from plants [5]. Cotton and jute are hugely produced all of total cellulosic fibres throughout the world. Cellulose is the basic structural component of all plant fibres whereas 61–71 % cellulose in jute fibre and 95–99 % cellulose in cotton fibre [6,7]. The cellulose content is one of the responsible for mechanical properties which high cellulose and low micro-fibril angle conveyed excellent mechanical properties [8]. Jute fibre is cheaper than the cotton fibre. To ensure the best quality while minimizing costs, jute-cotton composite fabric is created with cotton yarn in the warp and jute yarn in the weft, striking a balance between excellence and affordability [9].

Polymer composite is consist of matrix (thermoset or thermoplastic) and reinforced fibres (natural or synthetic) where the fibres are load bearing members and matrix material is bind and protect the fibres [10]. Unsaturated polyester resin is extensively utilized as a matrix material because of its numerous advantages, including its ability to cure at room temperature, excellent mechanical properties, low viscosity, affordability, and transparency [11]. The polymers are selected for suitable applications based on the properties such as physico-mechanical and thermal properties and chemical interaction. Aloe Vera gel serves as a natural plasticizer, primarily enhancing the flexibility and processability of polymers [12]. Aloe Vera gel (AV) is known to be a natural material and obtained from the leaves of AV plant which a member of the Liliaceae family. The major components of AV gel are composed of about 98.5–99.5 % water and the remaining solid materials more than 200 different components have been identified including polysaccharides, enzymes, fat-soluble vitamins, minerals, sterols, phenolic compounds and organic acids etc. [13,14]. The polysaccharides most abundant type of compounds among the remaining 0.5–1.5 % ingredients which are used as additives in the food industry and in many technical applications [15,16]. Natural fibres-based reinforced thermoset composites are extensively used in various fields of the domestics, technical fields and automobile sectors of light and heavy applications such as corrugated sheet, door panels, headliners, package trays, seat backs, seat backs, dashboards, and various interior components of car etc. [3].

Unsaturated polyester resin makes inherently brittleness due to its high cross-linking degree, which limit their applications in some field has been widely reported [17]. H. N. Dhakal and S. O. Ismail noted that elevating cross-link density results in enhanced strength, stiffness, chemical resistance, and glass transition temperature (*T*_*g*_), while simultaneously reducing strain at break [18]. R. Sultana et al. and N. E. Marcovich et al. reported that the unsaturated polyester resin exhibit brittleness and low elasticity [19,20]. Unsaturated polyester resin jute-cotton union fabric reinforced corrugated sheet cracks easily when iron nails are inserted. J. Diani and K. Gall reported that important drawback of unsaturated polyester resin being of the high crosslink density as it results in brittleness [21]. AVG-based composites have demonstrated huge potential for suitable applications owing to their plasticizer properties and reduced to brittleness [12]. Even though extensive research on the jute-cotton union fabric-reinforced unsaturated polyester resin composites has been conducted [22,23], but the integration of AVG into the unsaturated polyester resin jute-cotton union fabric-reinforced corrugated sheet and its effect have not been investigated.

In the current investigation, the jute-cotton union fabric-reinforced unsaturated polyester resin corrugated sheets are being produced by incorporating AVG through the application of the hand lay-up technique. The influence of AVG with different ratios like as 1 %, 3 %, 5 %, and 7 % on the water absorption behavior, degradation, physico-mechanical and thermal properties were investigated compared to without AVG based composite. Experimental investigations were conducted in this study to evaluate the tensile, flexural, cracking test, FT-IR spectroscopy, TGA analysis, thermal conductivity, water absorption behavior and degradation.

## 2 Materials and methods

### 2.1 Materials

**Fig. 1:**
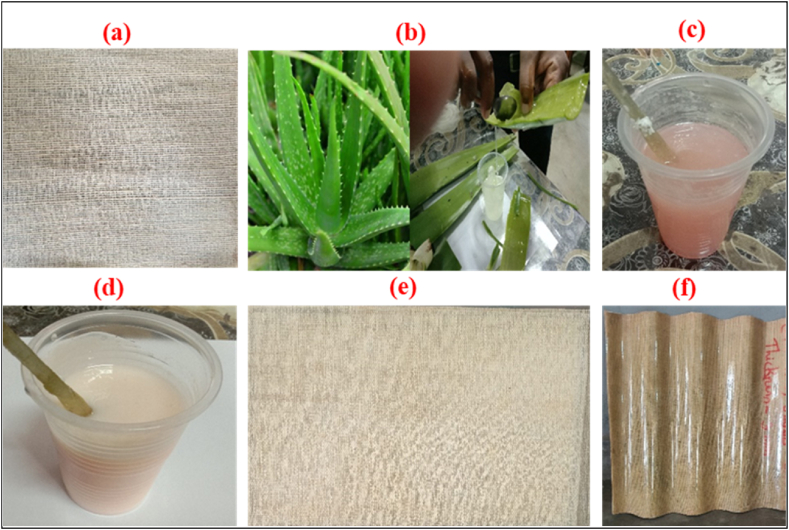
(a) Jute-cotton union fabric, (b) Aloe vera gel extract from aloe vera plant leaf, (c) Unsaturated polyester resin, (d) UPR, MEKPO, and AVG mixed resin, (e) Plain composite sheet, (f) Corrugated composite sheet.

During the experimental phase of this research, various raw materials such as jute-cotton union fabric, AVG, UPR, and hardener were utilized. Jute-cotton union fabric was collected from Bangladesh Jute Research Institute, Dhaka-1207, Bangladesh. The cotton and the jute yarn are used in the warp and weft direction respectively. Fabric specifications warp count, weft count, EPI, PPI, and GSM are 320 tex, 229 tex, 50, 16, 228, respectively. General purpose unsaturated polyester resin of orthophthalic grade was used. Trade Name “SHCP 268 BTQN” supplied by Singapore Highpolymer Chemicals Products Pte Ltd. (imported by Nasim Plastic Ltd., Bangladesh). Methyl ethyl ketone per oxide (MEKPO) and AVG were used as a catalyst. MEKPO was purchased from PT. KAWAGUCHI KIMIA INDONESIA. AVG was extracted by manually from the leaves of aloe vera plant which was collected in local market in Bangladesh, as shown in Fig. 1.

### 2.2 Fabrication of composite

**Fig. 2:**
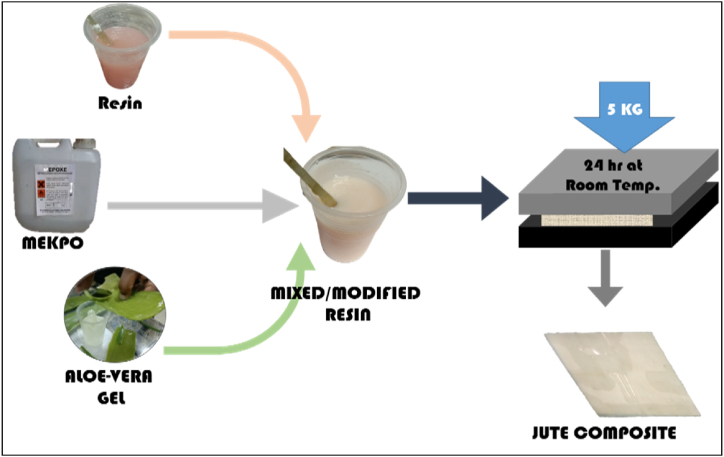
Fabrication process of the specimens.

In this study, the composites were made using the hand lay-up method, with the procedure depicted in Fig. 2. The matrix of unsaturated polyester resin and different percentages of AVG (1 %, 3 %, 5 %, and 7 % on the weight of UPR) are mixed thoroughly. Then the MEKP (3 % on the weight of UPR) catalyst was added in the mixture solution and made uniform mixture by stirring. Upon mixing the resin and aloe vera gel, the mixture's color transformed to a dense milk hue with a slight pink glow. The jute cotton union fabric is added layer by layer to matrix mixture, which were placed in the milot paper. The excess matrix materials were removed by pressing the hand roller. All the stacked materials were placed between two stainless steel and pressured by 5 kg weight kept curing for 24 h at room temperature [24,25]. After the curing the composite was released from mould and are cut to prepare test specimens.

### 2.3 Fourier transforms infrared spectroscopy (FTIR)

FTIR examinations were conducted on UPR composites reinforced with jute-cotton union fabric and incorporating varying percentages of AVG. The analyses were conducted utilizing an FT/IR – 4600LE JASCO instrument in attenuated total reflectance (ATR) mode, employing a diamond crystal. The samples underwent testing within a transmittance range of 500–4000 cm^−1^, employing 16 repetitions per scan average for each sample and a resolution of 4 cm^−1^.

### 2.4 Cracking test

An experiment was manually conducted to determine the effect of AVG as a plasticizer in the produced sample. A 2-inch iron nail has been tapped against both without AVG and AVG based composite sample with a hammer of 250 g weight by hand. The hammering was done at medium pace from approximately 2-inch perpendicular distance. Several strokes are needed until the nail finds its way out approximately 10 mm in the other side.

### 2.5 Thermal conductivity test

The materials for which thermal conductivity is to be determined should be cut into test specimens measuring 20.6 mm in diameter and 3 mm in thickness. Thermal conductivity of the composites was measured using Modified Lees’ disk apparatus with ASTM D-7340. The thermal conductivity was calculated using equation (1):

(1) $$\text{Thermal}\;\text{Conductivity}\;\left(K\right)=\frac{ed}{2\pi r^{2\;\left(T_{B}-T_{A}\right)}}\;\left({2a}_{A}T_{A}+a_{s}\frac{T_{A}+T_{B}}{2}\right)$$

### 2.6 Mechanical tests

The tensile and flexural properties of the developed composites were assessed utilizing the Hounsfield series S testing machine (UK), employing a cross-head speed of 1 mm/s and a gauge length of 20 mm. The test specimens were cut to the required dimension using a band saw, following the guidelines of ASTM-D638-01. It is important to note that all test values were obtained as the average of three samples.

### 2.7 Thermal analysis

The thermal stability of the samples was assessed using a thermogravimetric analyzer (ELTRA THERMOSTEP, Germany) following the ASTM D7348 – 08e1 standard. A minimum of 400 mg of the composite specimen was loaded into an alumina crucible for testing, and the procedure was conducted under an inert atmosphere with nitrogen gas. The temperature was incrementally raised at a rate of 5 °C/min from room temperature to 600 °C. Weight loss percentage was recorded as a function of temperature.

### 2.8 Water absorption tests

The water absorption behavior on unsaturated polyester resin composites reinforced with jute-cotton union fabric and incorporating varying percentages of AVG was investigated following ASTM D 570 standards [26]. The samples were immersed in a sea and distilled water bath at room temperature for 1 day to 15 days. To measure water absorption, the samples were removed from the water, wiped dry with a clean cloth, and then weighed using a precision electronic balance accurate to 10^−4^ gm. The weight difference was used to calculate the percentage change in weight according to equation (2).

(2) Percentagechangeinweight=Weightdifference/Initialweight×100

### 2.9 Degradation test

The degradation of the developed AVG and non-AVG based composite was investigated by subjecting them to sodium hydroxide (NaOH) and hydrochloric acid (HCl). The purpose of this investigation is to study the degradation behavior of these composites under varying pH conditions. To conduct the study, sample size of 1.5 × 1.5 × 0.3 cm was carefully weighed and then immersed in the respective solvent for a duration of 15 days at room temperature. The specimens were taken out, cleaned, and weighed. The weight loss (%) of the specimens were calculated according to equation (3).

(3) Theweightloss(%)=Finalweight/Initialweightx100

### 2.10 Soil burial

To assess how soil burial affects the biodegradability of composite specimens, a biodegradation test was conducted using the soil burial method for a period of 10 weeks. The experiment occurred in a glass beaker filled with farmland soil, and water was periodically sprinkled. The composite specimens were placed in the soil at a depth of 3 cm from the surface, with an average temperature of 27 °C maintained consistently during the exposure period. After the 10-week period, the specimens were retrieved from the soil, washed with tap water, and subsequently dried at 100 °C in an oven for 1 h [27,28]. Biodegradation behavior was assessed by measuring weight loss.

## 3 Results and discussion

### 3.1 FTIR analysis

**Fig. 3:**
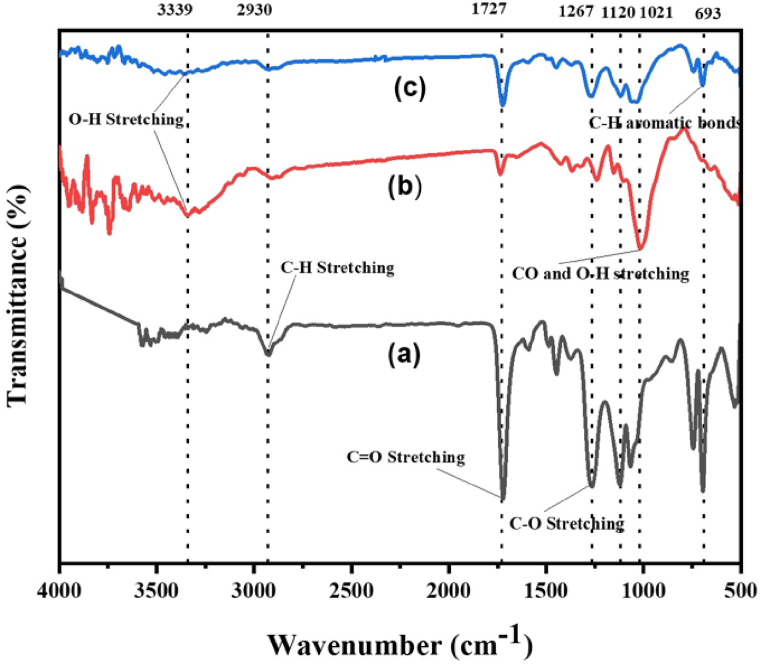
(a) Cured polyester resin (b) Jute cotton union fabric (c) AVG incorporation into UPR jute cotton union fabric reinforced composite.

Fig. 3 displays FTIR spectra for the cured polyester resin, the jute-cotton union fabric, and the composite material with AVG incorporated into UPR jute-cotton union fabric.

**Fig. 4:**
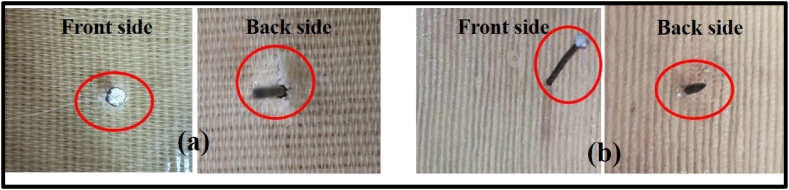
(a) Without AVG composite, (b) AVG based composite.

The spectra cover the entire absorption range, revealing the distinctive functional groups corresponding to each absorption band. The peak observed at 3339 cm^−1^ represents the distinctive band associated with hydroxyl groups, indicating the stretching vibration of the O–H group [29,30].The bands detected at 2930 cm^−1^ can be attributed to the stretching vibrations of CH groups, including CH_2_ and CH_3_. The observed reduction in peak intensity when AVG is incorporated into UPR jute-cotton union fabric-reinforced composite, as opposed to the cured polyester resin, suggests a hydrogen bond interaction among jute-cotton union fabric, AVG, and UPR [31,32]. In the spectra of the cured polyester resin, jute-cotton union fabric, and jute-cotton union fabric reinforced with AVG incorporation, a prominent band at 1727 cm^−1^, associated with the stretching vibrations of the C=O group, was distinctly observed [33,34]. Furthermore, when AVG is incorporated into UPR jute-cotton union fabric-reinforced composite, the peak intensity at 1727 cm^−1^ is decreased compared to the cured polyester resin. The peak at 1267 cm^−1^ confirms to the stretching vibration of CH_2_ groups [29,35]. The peak intensity of AVG-based composites is decreased in contrast to cured polyester resin, which could be related to functional group conversion during the blending polymer process. The band at 1120 cm^−1^ which attributed to the C–O stretching vibration of C–C linkages [36]. CO and O–H stretching vibration peak of 1021 cm^−1^, whereas the peak at 693 cm^−1^ in the fingerprint region indicates the presence of benzene [29,37]. It could be concluded that the characteristics of its peak, peak intensity and shape are varied due to interactions and change occurring at the molecular level between the AVG and UP resin jute-cotton union fabric composites.

### 3.2 Cracking analysis

Fig. 4 illustrates the observation of both the front and back side of the AVG-based and non-AVG composites. In the case of the jute cotton union fabric reinforced composite without AVG, there are some scratches and fiber displacement noticed around the nail. There has been a prominent fracture in the back side of the composite without AVG. Unsaturated polyester resin has high cross linking density resulting brittleness [38]. On the other hand, the composite sample produced with AVG shows a significant improvement on both sides. The front side of the composite sample produced with AVG has no trace of fracture or scratch. However, it has very little stretch mark on the backside. Aloe Vera gel serves as a natural plasticizer, primarily enhancing the flexibility and processability of polymers [12,39,40]. This experiment proves the practical effect of AVG as plasticizer in the sample composite.

### 3.3 Thermal conductivity analysis

Table 1 displays a comparison of the thermal conductivity among various composite samples with differing percentages of AVG, as well as samples without AVG.

**Table 1:** Results of thermal conductivity of the specimens.

Sl. No.	AVG (%)	Thermal Conductivity (Wm^−1^K^−1^)	Standard Deviation
1.	0 %	0.2943081	0.014715405
2.	1 %	0.2852528	0.011410112
3.	3 %	0.2735385	0.019147695
4.	5 %	0.2649139	0.015894834
5.	7 %	0.2601830	0.00780549

The thermal conductivity of a material indicates the quantity of energy conducted through a unit area and unit thickness of the material in a given time, when there's a unit temperature difference between the faces causing heat flow [41]. The thermal conductivity of jute cotton union fabric reinforced composites with 1 %, 3 %, 5 %, and 7 % of AVG incorporated are 0.295, 0.285, 0.273, 0.265, and 0.260 Wm^−1^K^−1^ respectively. The thermal conductivity of the jute cotton union fabric composite without AVG is found to be 0.294 Wm^−1^K^−1^. It can be noticed that the increase in AVG percentage incorporated in the jute cotton union fabric reinforced composite has caused a decrease in thermal conductivity values. Table 1 shows that the jute cotton union fabric composite, manufactured with 7 % AVG, exhibits the lowest thermal conductivity among all composites. Aloe Vera Gel is primarily made up of water, polysaccharides, and a range of organic compounds, all of which inherently exhibit low thermal conductivity. When incorporated into the resin matrix, AVG contributes its thermal insulating characteristics to the composite [14,42].

## 4 Mechanical properties

### 4.1 Tensile strength and modulus

**Fig. 5:**
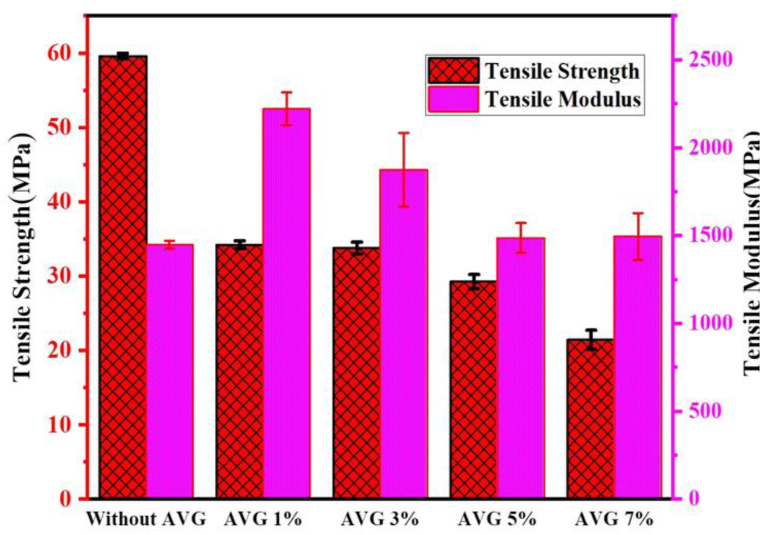
Tensile strength and modulus.

The comparison of mechanical properties of the developed composite containing different percentages of AVG are shown in Fig. 5.

The findings indicate a noticeable decline in tensile strength and modulus as the concentration of aloe vera gel is increased from 1 % to 7 % in the unsaturated polyester resin (UPR) jute cotton union fabric reinforced composite (Fig. 5). The composite lacking AVG exhibits the highest tensile strength, measuring at 59.59 MPa, whereas the composite containing 7 % AVG demonstrates the lowest tensile strength, measuring at 21.43 MPa. The uniformity of the pattern in tensile modulus displays some variability. The composite with 1 % AVG showcases the highest tensile modulus at 2221.6 MPa, while the composites with 3 % and 5 % AVG exhibit lower tensile moduli when compared to the composite lacking AVG. The incorporation of AVG appears to adversely affect the tensile characteristics of the composite. Increasing AVG% leads to a more pronounced decline in tensile strength. The decline in tensile strength as the AVG% increases may be attributed to factors like reduced cross-link density and poor adhesion between the AVG and the composite matrix, resulting in a less efficient transfer of loads [43].

### 4.2 Flexural strength and modulus

**Fig. 6:**
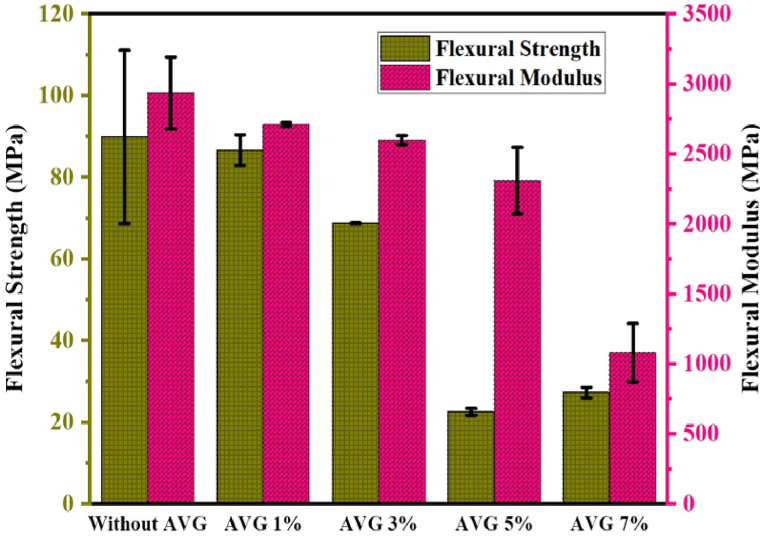
Flexural strength and modulus.

The flexural properties of the UPR jute-cotton union fabric composites with different percentages of AVG are shown in Fig. 6. The findings suggest that the mechanical properties of the composite, specifically the flexural strength as well as flexural modulus, are affected by the concentration of AVG. Generally, higher concentrations of AVG tend to result in reduced mechanical properties as shown in Fig. 5, Fig. 6. The flexural strength of the composite is measured at 89.8485 MPa when AVG is not present. Nevertheless, as the concentration of AVG rises from 1 % to 7 %, there is a progressive decline in flexural strength. The concentration of AVG also plays a role in influencing the trend of flexural modulus. The composite without AVG displays the highest flexural modulus, measured at 2934.2105 MPa. However, the inclusion of AVG results in a decline in flexural modulus. The inclusion of 1 % AVG in the composite results in a slight reduction in both flexural strength and flexural modulus. This implies that using a lower concentration of AVG may have a less significant effect on these properties [44].

### 4.3 Thermal analysis

**Fig. 7:**
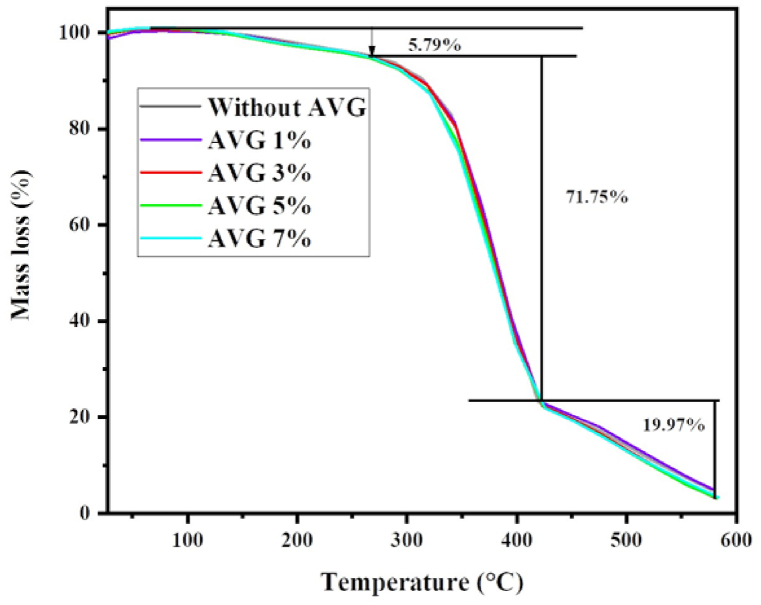
TGA curves of with and without AVG based composites.

Fig. 7 depicts the variation of thermal decomposition for composites containing varying percentages of AVG over temperature range from room temperature to 600 °C. The influenced the thermal degradation by the polymer blend interaction as well as cross links formed. The thermal decomposition process of the AVG-based composites results in three pertinent ranges in the analysis. The first temperature range was observed between room temperature and 115 °C, with minor weight loss observed due to evaporation of the composites’ moisture content [45]. Between 260 °C and 420 °C, the temperature range showed the second most notable weight loss. This major weight loss happened due to the decomposition of the polymer backbone, cellulose, and hemicellulose [[46], [47], [48]]. The composite with 1 % AVG showcases higher thermal stability compared to other samples. But in general, the increased incorporation of AVG into the unsaturated polyester resin matrix, the decrease the thermal stability as evidenced by the TGA analysis in Fig. 7. The conclusion from the analysis was that AVG-based composites have low thermal stability when compared to the control sample due to the presence of huge amount water content in the AVG and reduced the cross-link density.

### 4.4 Water absorption properties

**Fig. 8:**
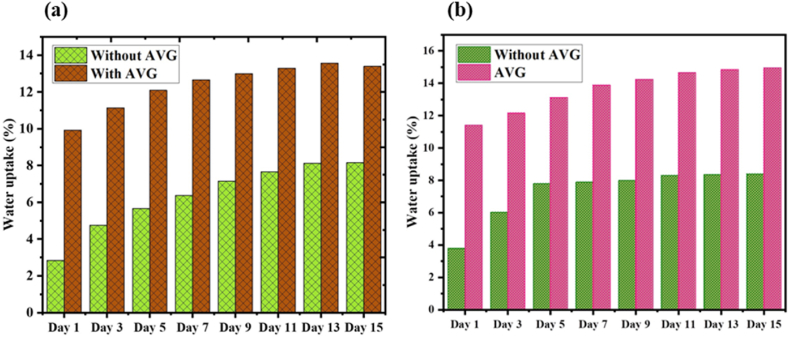
(a) Sea and (b) Distil water absorption behavior of the with and without AVG based composite.

The effect of adding AVG into the jute-cotton union fabric reinforced composites was examined and presented in Fig. 8. The results presented demonstrate the water absorption characteristics of the composite material in the presence and absence of AVG. These findings suggest that the inclusion of AVG can potentially impact the material's interaction with water during the specified duration. Water absorption in both composites (with and without AVG) shows an increase over the 15-day duration.

The composite containing AVG consistently demonstrates higher percentages of sea and distil water uptake compared to the composite without AVG at each corresponding time interval. This trend indicates that the inclusion of AVG potentially enhances the composite material's ability to absorb water [49].

### 4.5 Solvent resistance or degradation analysis

**Fig. 9:**
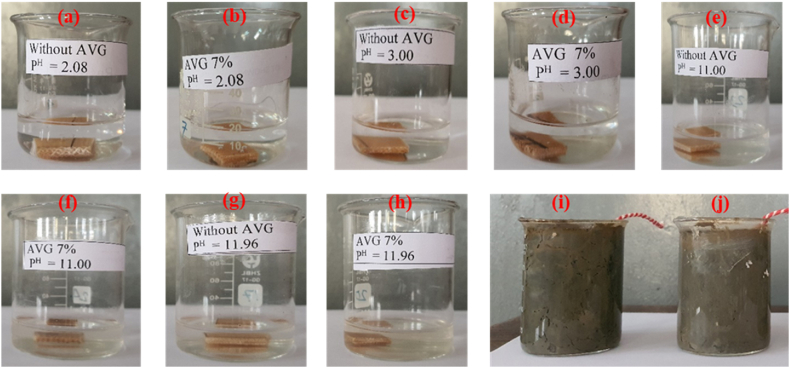
(a–d) Acid degradation, (e–h) alkali degradation and (i–j) soil degradation behavior of the with and without AVG based composites.

Solvent resistivity is a crucial factor to take into account when using cured unsaturated polyester resin in their final applications. The measurement of the degradation rate of composite specimens subjected to various environments, like acid, alkali, and soil burial, was conducted by assessing the weight loss percentages of the specimens prior to and after the exposure as shown in Table 2 and Fig. 9.

**Table 2:** Chemical resistance and biodegradable test of with and without AVG based composite.

Sample Name	Alkali test		Acid test		Soil test
	P^H^ = 11.00	P^H^ = 11.96	P^H^ = 2.08	P^H^ = 3.00	
Without AVG based composite	101.07 %	102.12 %	101.97 %	101.43 %	100.10 %
AVG based composite (AVG 7 %)	98.84 %	97.78 %	98.26 %	98.56 %	97.62 %

The evaluation was conducted to determine the percentage of weight change for the AVG based partial biocomposite in alkali (P^H^-11.00 and P^H^ -11.96) and acid (P^H^ -3.00 and P^H^ -2.08) [50]. This experiment finds that AVG-based partial biocomposite loses weight for both alkali and acid media.

The weight of the specimen showed a slight decrease, with the AVG composite specimen buried in the soil experiencing the highest weight loss of 2.38 %. This could be attributed to the surface attack of bacterial microbes present in the moist soil on the composite. Additionally, the fibers on the specimen's surface may have absorbed moisture from the soil, causing fiber swelling and resulting in the debonding of the fiber matrix.

## 5 Conclusion

In this study, composites were fabricated using jute-cotton union fabric reinforced unsaturated polyester resin, incorporating varying amounts of AVG. The characteristics of these composites were investigated in relation to the different quantities of AVG present. The incorporation of AVG increases from 1 % to 7 % in the composites, leading to a gradual decrease in composite thermal conductivity. This indicates that the material functions as a good insulator. When iron nails are inserted, the AVG-based composite shows no signs of scratches or fiber displacement. However, the increase in AVG resulted in a reduction of cross-link density in the AVG-based composite, leading to a decrease in mechanical properties and an increase in thermal degradation. The incorporation of AVG up to 7 % in the composites increases water solubility and degradation rate. Therefore, jute-cotton union fabric reinforced unsaturated polyester resin composites with AVG can be utilized as insulation material or corrugated sheets for tropical country/rural poultry housing.

## Data availability statement

Data will be made available on request.

## CRediT authorship contribution statement

**Md Anisur Rahman Dayan:** Writing – original draft, Methodology, Investigation, Formal analysis, Conceptualization. **Md Mahmudul Habib:** Writing – review & editing, Validation, Data curation. **Md Moslem Uddin:** Writing – review & editing, Visualization, Investigation. **Mahmuda Khatun:** Validation, Investigation, Data curation. **Md Sahadat Hossain:** Writing – review & editing, Validation, Methodology, Investigation. **Muhammad Abdur Rashid:** Writing – review & editing, Validation, Supervision, Resources, Conceptualization.

## Declaration of competing interest

The authors declare that they have no known competing financial interests or personal relationships that could have appeared to influence the work reported in this paper.
